# Preparation of Solid Dispersions of Simvastatin and Soluplus Using a Single-Step Organic Solvent-Free Supercritical Fluid Process for the Drug Solubility and Dissolution Rate Enhancement

**DOI:** 10.3390/ph14090846

**Published:** 2021-08-25

**Authors:** Uttom Nandi, Adejumoke Lara Ajiboye, Preksha Patel, Dennis Douroumis, Vivek Trivedi

**Affiliations:** 1 Medway School of Pharmacy, University of Kent, Central Avenue, Chatham Maritime, Chatham, Kent ME4 4TB, UK; U.Nandi@kent.ac.uk (U.N.); L.Ajiboye@kent.ac.uk (A.L.A.); 2 School of Science, University of Greenwich, Central Avenue, Chatham Maritime, Kent ME4 4TB, UK; preksha0296@gmail.com (P.P.); D.Douroumis@greenwich.ac.uk (D.D.)

**Keywords:** simvastatin, supercritical carbon dioxide, soluplus, drug dissolution

## Abstract

The study was designed to investigate the feasibility of supercritical carbon dioxide (scCO_2_) processing for the preparation of simvastatin (SIM) solid dispersions (SDs) in Soluplus^®^ (SOL) at temperatures below polymer’s glass transition. The SIM content in the SDs experimental design was kept at 10, 20 and 30% to study the effect of the drug–polymer ratio on the successful preparation of SDs. The SIM–SOL formulations, physical mixtures (PMs) and SDs were evaluated using X-ray diffraction (XRD), differential scanning calorimetry (DSC), attenuated total reflectance-Fourier transform infrared spectroscopy (ATR-FTIR), scanning electron microscopy (SEM), and dissolution studies. The scCO_2_ processing conditions and drug–polymer ratio were found to influence the physicochemical properties of the drug in formulated SDs. SIM is a highly crystalline drug; however, physicochemical characterisation carried out by SEM, DSC, and XRD demonstrated the presence of SIM in amorphous nature within the SDs. The SIM–SOL SDs showed enhanced drug dissolution rates, with 100% being released within 45 min. Moreover, the drug dissolution from SDs was faster and higher in comparison to PMs. In conclusion, this study shows that SIM–SOL dispersions can be successfully prepared using a solvent-free supercritical fluid process to enhance dissolution rate of the drug.

## 1. Introduction

Currently, one of the major issues limiting the biological application of a number of active pharmaceutical ingredients (APIs) is undoubtedly linked to their low aqueous solubility. It is estimated that as many as 70% of APIs and new clinical entities have poor water solubility, which leads to a slow absorption, and an inadequate and variable bioavailability of the drug [1,2]. Approaches to improve the dissolution properties of poorly aqueous-soluble drugs include: particle size reduction, salt formation, cocrystallisation and the use of surfactants and co-solvents [3]. However, each of these techniques still has their own practical limitations; for example, the difficulty in salt formation for neutral and weakly acidic/basic drugs, while the use of surfactants/co-solvents results in liquid formulations that are known to have reduced commercial viability and patient tolerability. The particle size reduction methods are highly energy intensive and can lead to the formation of fine powders that have low wettability and a high tendency to form agglomerates [3,4]. Therefore, there is an imminent need to develop alternative solubility enhancement techniques such as the development of solvent-free preparation of SDs to help overcome the aforementioned limitations.

A solid dispersion (SD) comprises of a minimum of two different components, usually a hydrophilic matrix and a hydrophobic drug [5]. The drug in these systems is distributed within the crystalline or amorphous matrix, either molecularly or as particulates. A solid solution with a molecularly dispersed drug is of particular interest as it can result in dissolution rate enhancement owing to the decrease in drug crystallinity and an increase in the specific surface area [5,6]. Generally, techniques used to obtain SDs include: spray-drying [7], co-evaporation or co-precipitation [8], freeze-drying [9], and hot-melt extrusion (HME) [10,11]. However, each of these processes has its own disadvantages that might restrict its use. HME is extremely popular in SD preparation but the possible thermal degradation of drugs and/or polymers caused by the high processing temperatures can be considered as a principle drawback of this technique [12]. Similarly, other conventional techniques may require either one or more combinations of organic solvents, shear stress and high temperatures that can limit yield and lead to chemical, thermal or shear-induced product degradation. Moreover, the use of organic solvent requires additional drying steps, which adds to the production cost, and residual solvent toxicity in the formulation always remains a realistic concern [12,13,14]. Therefore, it is important to explore other processing techniques such as supercritical fluid (SCF) processing, which may be capable of avoiding many of the abovementioned drawbacks [13,14,15].

SCF can be defined as a substance above its critical pressure and temperature, where it possesses properties of both liquids and gases (density similar to liquids, whereas the diffusivity and viscosity are akin to gases) [16]. The use of SCF-based methods for the formulation of SDs can result in end products with narrow particle size, better flowability and lower or no residual solvent content [17]. There are numerous SCFs, but scCO_2_ is preferable in the processing of organic compounds due to its low critical temperature (31.1 °C) and pressure (73.8 bar). CO_2_ is also non-toxic, inert and cheap in comparison to many organic solvents that can be used as SCF [18,19]. Moreover, tuneable properties of scCO_2_ make it extremely versatile in the pharmaceutical processing as it can be applied as an anti-solvent, extraction agent, solvent and/or plasticizer for numerous amorphous or crystalline drugs and polymers. Additionally, the easy separation of CO_2_ from the polymer matrix at the end of the formulation process ensures that only solvent-free products are produced [12,13,17,19]. SDs of various drugs prepared by scCO_2_ processing have already been reported in literature, as presented in Table 1.

**Figure 1:**
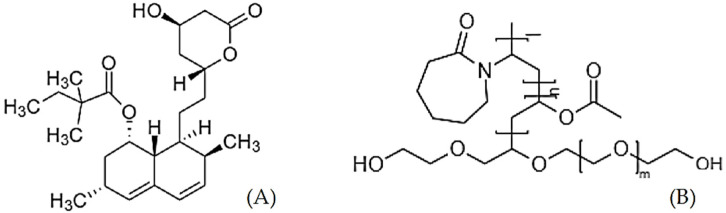
The structure of Simvastatin (**A**) and Soluplus (**B**).

In this work, a single-step scCO_2_ processing method was developed for the preparation of SDs of SIM, using SOL as the hydrophilic carrier. SIM (Figure 1A) is one of the most commonly prescribed cholesterol and lipid-lowering agents available in the market [36]. SIM is a crystalline API with the molecular mass of 418.56 g/mol and classified as a class II drug according to the Biopharmaceutics Classification Scheme (BCS) [37,38]. Hence, its dissolution rate plays a crucial role in attaining the desired drug level in the systemic circulation for a biological response [37,38]. Several methods have been investigated to improve the solubility of SIM, with the preparation of SD proving to be the most promising in terms of ease and efficiency [37].

SOL (Figure 1B) is a novel amorphous, amphiphilic, co-polymer comprised of polyvinyl caprolactam, polyvinyl acetate, and polyethylene glycol [39]. Unlike conventional solubility enhancers like Cremophor^®^ RH 40, the bifunctional SOL is able to improve the dissolution rate of poorly soluble drugs through the formation of micelles and as a polymer matrix in SDs [40]. The non-ionic and hydrophilic properties of SOL, along with its slightly surface-active characteristics, help in sustaining the solubility of a loaded hydrophobic drug along with maintaining the drug supersaturation throughout the gastrointestinal tract [39,40]. SOL has been proven to enhance the dissolution rate of BCS class II drugs, including SIM by the fabrication of SD via HME [38]. However, the SIM–SOL amorphous dispersion prepared with HME required high working temperatures of up to 150 °C. scCO_2_ has been used in the micronisation of SIM [41], but, to our knowledge, there are no reports on the development of SD via scCO_2_ technology using SOL as a carrier matrix. Hence, the aim of this work was to formulate SIM–SOL SDs without the aid of any organic solvent via scCO_2_ processing at low temperatures to improve the dissolution rate of SIM.

## 2. Results and Discussion

The affinity of both drug and selected carrier towards scCO_2_ is the most critical aspect in the successful preparation of SDs via the SCF processing method [39]. Typically, the successful formation of SD from the drug–polymer mixture in scCO_2_ involves key steps, including (a) drug–polymer liquid/melt generation, (b) mixing of both at the liquid/molten state, and (c) their subsequent solidification. The lowest pressure and temperature for the solid–liquid (S–L) transition of SIM were 120 bar and 50 °C in scCO_2_. The increase in pressure to 200 bar did not result in the further reduction of S–L transition temperature. Although, the phase change of SOL in scCO_2_ can be obtained at subcritical conditions (30 °C/82 bar) [38,39], but these were considered impractical processing conditions in this case as they are lower than the one determined for SIM. Hence, a number of experiments were designed to investigate the effect of temperature, pressure, duration, and drug/polymer ratio, as shown in Table 2.

In each case, temperatures and pressures were kept above the S–L transition values of the polymer, but not necessarily of the drug’s, to understand if it was necessary for both SOL and SIM to be in molten state for the successful formation of SDs. The preparation of SDs at the conditions listed in Table 2 suggested that the minimum pressure, temperature, and duration to obtain completely amorphous systems were 150 bar, 50 °C and 120 min, respectively. scCO_2_ processing at 40 °C always resulted in formulations with SIM in crystalline state, irrespective of the drug/polymer ratio, pressure, and duration. Hence, it was concluded that both SIM and SOL need to be in molten state to obtain SDs successfully in scCO_2_. SD prepared with 30% SIM was also semi-crystalline, which was likely due to the presence of higher drug content in the formulation.

### 2.1. DSC Analysis

**Figure 2:**
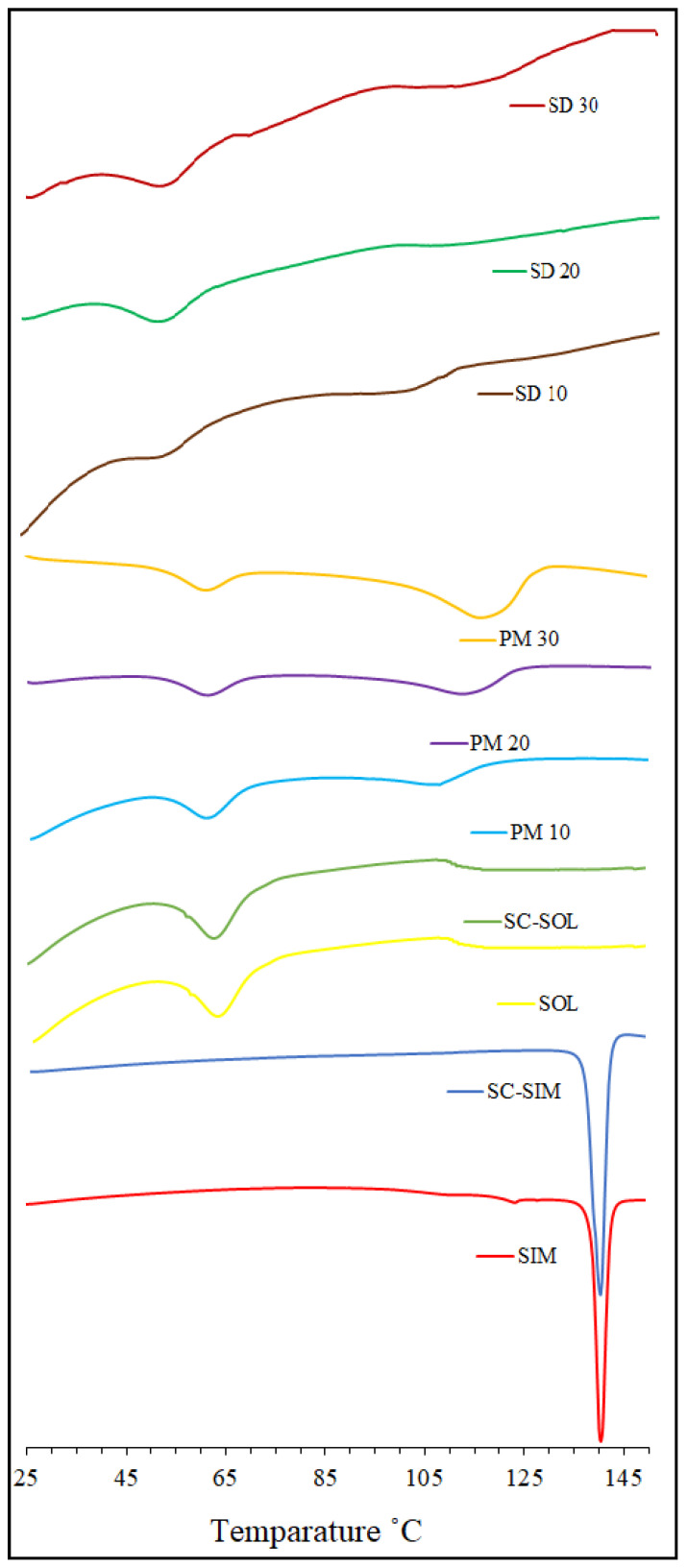
DSC thermogram of Soluplus^®^, Simvastatin, physical mixture (PM10, PM20, and PM30 and solid dispersions (SD10, SD20, and SD30) prepared at 50 °C and 150 bar.

DSC analysis was performed to investigate the physical state of SIM within the polymer matrix after scCO_2_ processing. Thermograms for SIM, scCO_2_ processed SIM (SC-SIM) and SOL (SC-SOL), along with the formulations (PMs and SDs prepared at 50 °C and 150 bar), are presented in Figure 2. The DSC curve for SIM and SC-SIM showed a sharp melting peak at around 140 °C due to its characteristic crystalline structure [42]. On the other hand, SOL as an amorphous polymer presented a broad endothermic event between 56 and 70 °C, related to the glass transition temperature [39]. Another endothermic event was observed for SOL at 197 °C, which could suggest possible decomposition of the material.

The scCO_2_ processing of the polymer and drug alone did not show any changes to the characteristic thermal properties of SOL and SIM. The high-pressure treatment can have a strong impact on the crystalline structures in terms of polymorph formation and recrystallisation during the depressurisation step. The scCO_2_ processing of SIM did not result in any changes to its crystal habit, as evident from the thermograms in Figure 2. Moreover, a lack of sharp melting curve in SDs suggested that the drug did not recrystallise at the end of the processing. These observations, combined with the fact that no solvent or high temperatures were required to prepare these SDs, confirm the suitability of scCO_2_ processing as a method to prepare SIM–SOL SDs in these specified conditions.

The thermograms obtained for PMs showed a slight decrease in the polymer T_g_, while the SIM melting endotherm appeared broad and shifted to lower temperatures, varying from 110 °C to 120 °C. This indicates the high drug–polymer miscibility and at least partial dissolution of SIM in the polymeric melt. The SOL as a carrier has considerable low T_g_ compared to the melting temperature of SIM. Hence, SIM in the mixture can gradually dissolve in the polymer during the analysis and may not remain as a crystalline solid for a sharp melt peak to appear close to its melting point. For SDs, there was a shift in the polymer’s T_g_ to the lower temperatures, while the SIM melting endotherm was not detected. The absence of sharp melt peak of the drug suggests the existence of a glass solution where SIM is mostly dispersed in the amorphous polymer matrix and the T_g_ shifts can be attributed to the drug–polymer interactions.

### 2.2. XRD Analysis

**Figure 3:**
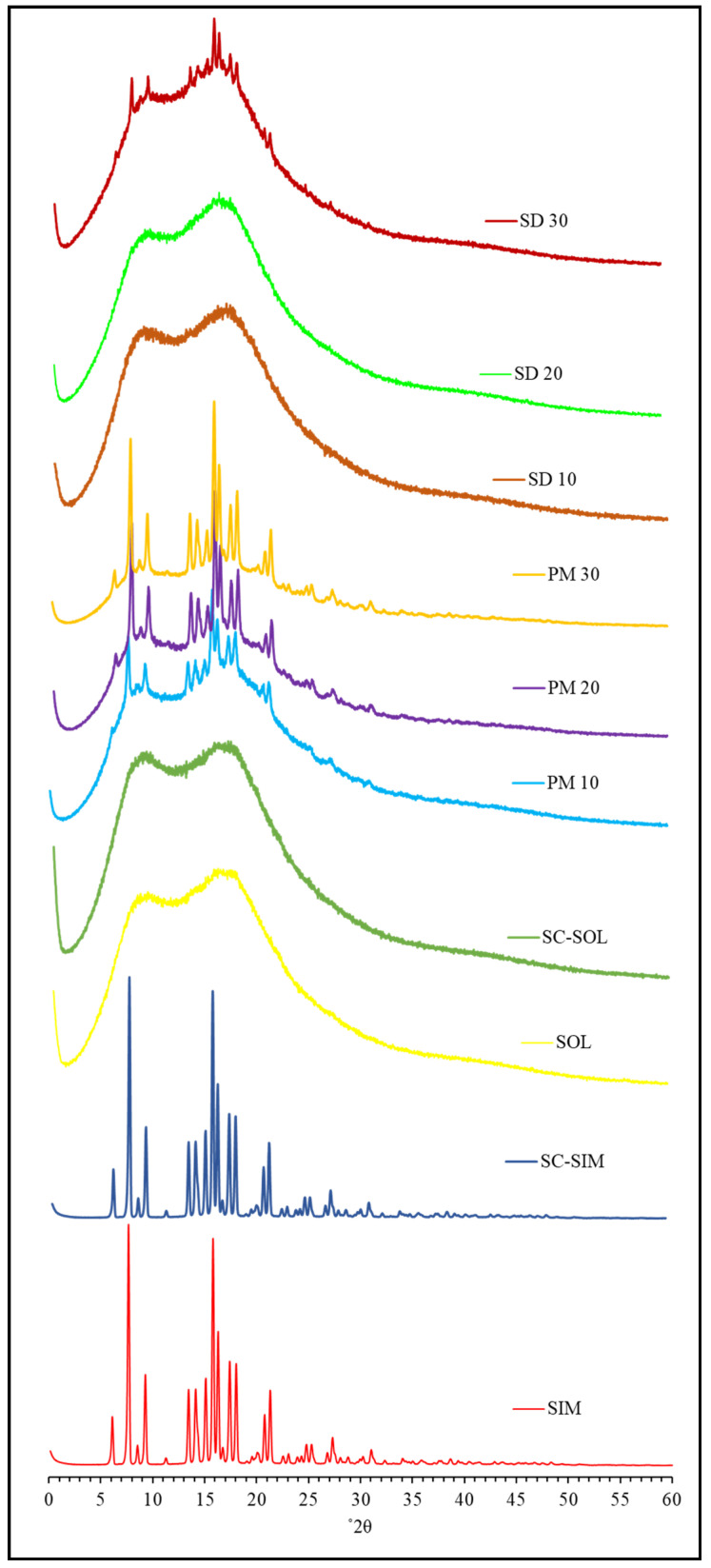
XRD diffractogram of Soluplus^®^, Simvastatin, physical mixture (PM10, PM20, and PM30 and solid dispersions (SD10, SD20, and SD30) prepared at 50 °C and 150 bar.

XRD analysis was performed to investigate the changes to the degree of crystallinity of the bulk API both in physical blends and the SDs prepared via scCO_2_ processing. The X-ray diffractograms of bulk and scCO_2_-treated SIM, SOL, and PMs and SDs are presented in Figure 3.

The XRD data obtained for SIM (raw and scCO_2_ processed) confirmed its crystalline structure with typical and sharp interference peaks at 2θ equal to 10.9°, 15°, 17.2°, 18.9°, 19.6°, and 22.1° [43]. The diffractogram of SOL displayed no peaks due to its amorphous nature. The three PMs (10, 20, and 30% *w*/*w*) and SD30 showed similar diffraction patterns to that of SIM but with reduced intensity of the diffraction peaks. In contrast, there was an absence of any sharp peaks in the diffractogram obtained for the SDs prepared with 10% and 20% SIM. The decrease or absence in crystalline peaks in diffractograms infers to the amorphous nature of SIM in SDs and confirms the result obtained by DSC.

### 2.3. ATR-FTIR Analysis

**Figure 4:**
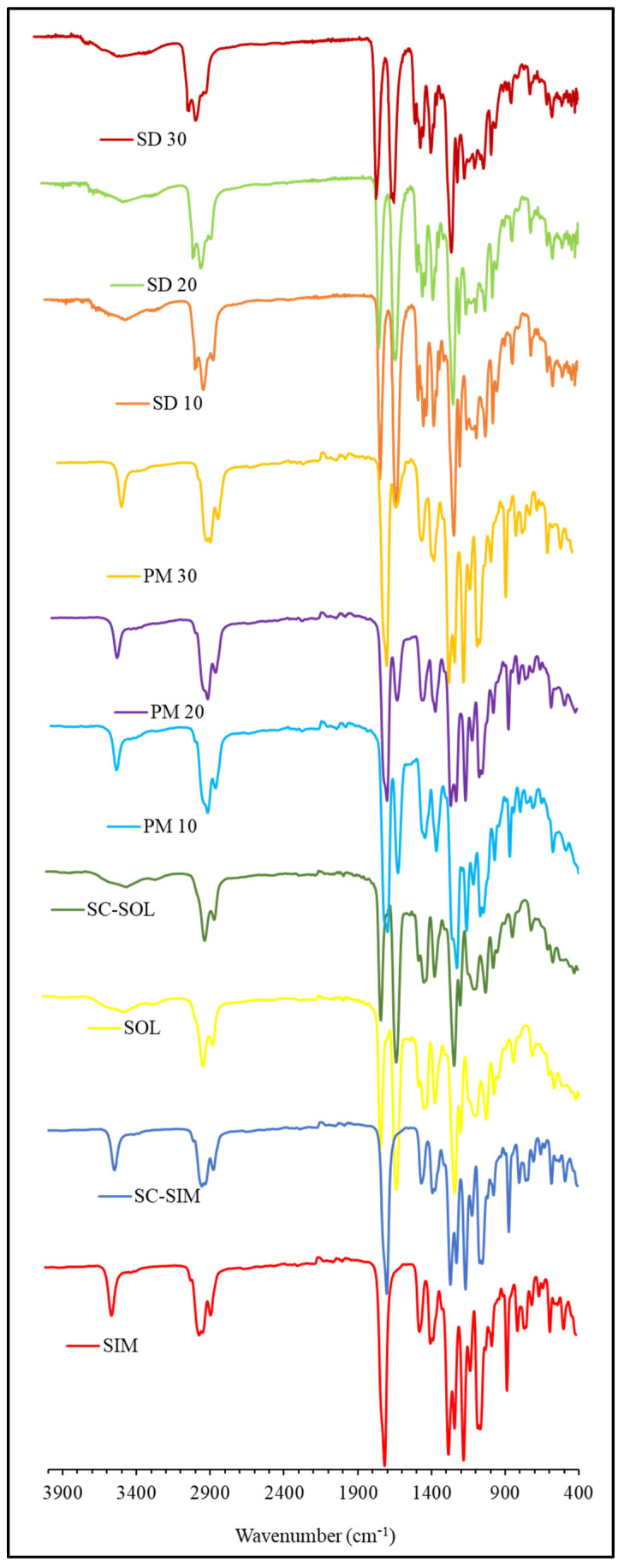
ATR-FTIR spectra of Soluplus^®^, Simvastatin, physical mixture (PM10, PM20, and PM30 and solid dispersions (SD10, SD20, and SD30) prepared at 50 °C and 150 bar.

The structural features of PMs and formulated SDs were studied by performing ATR- FTIR analysis on bulk and scCO_2_ processed drug and polymer and compared with PMs and SDs, as presented in Figure 4.

The characteristic absorption peaks of SIM were observed at 3548 cm^−1^ (free O–H stretching vibration), 2929 cm^−1^ (aromatic C-H stretching vibration), 1723 and 1695 cm^−1^ (stretching vibration of C=O for ester and lactone) [44]. In the case of SOL, the absorption peaks including a broad band at 3476 cm^−1^ (O-H stretching), 2924 cm^−1^ (aromatic C-H stretching), 1732–1625 cm^−1^ (C=O stretching), and 1478 cm^−1^ (C-O-C stretching) were observed in their ATR-FTIR spectrum [35,45]. The spectra collected for SDs and PMs overlap with that of unprocessed SOL, without displaying any significant peaks for SIM, thus indicating the presence of proportionally higher polymer content in the formulated systems. Although the characteristic peak for the drug at 3548 cm^−1^ (-OH stretching) is clearly visible at the same wavelength for all three PMs, it disappeared in the spectra of SDs. This absence or broadening of -OH stretching band has been reported before for the drug molecularly dispersed in a polymer that could be an evidence to a drug’s amorphous nature [12,13,14,34,35,46,47]. Interestingly, peak intensity of tertiary amide C=O stretching vibration of SOL at 1625 cm^−1^ expectedly reduced with the increasing ratio of SIM in the physical mixture but, in general, remained unaffected in the SDs. This could be attributed to the enhanced hydrogen bonding between the lipophilic portions of the drug and the polymer [48]. This was similarly observed in SIM/SOL amorphous solid dispersions prepared via hot-melt extrusion, as reported by Zhang et al. [27]. In addition, the stretching vibration of the ester carbonyl functional group showed a shift from 1732 to 1710 cm^−1^. These shifts of ester bond peak represent intermolecular hydrogen bonding, which is also in agreement with previously reported literature [38,49]. The FTIR results indicate favourable interactions between the drug and carrier. Moreover, drug amorphicity in SDs can be beneficial, as it usually indicates enhanced drug dissolution, whereas, surrounding polymeric matrix can prevent drug recrystallization [34].

### 2.4. SEM Analysis

**Figure 5:**
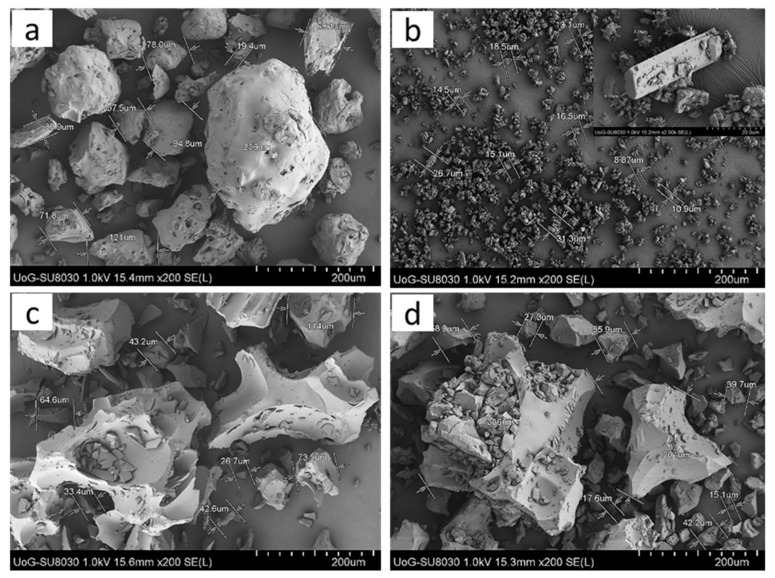
SEM micrographs (200×) of (**a**) Soluplus^®^, (**b**) Simvastatin (inset-2000×), and solid dispersions ((**c**) SD10 and (**d**) SD30).

The surface morphology of prepared SDs was investigated by SEM and compared to that of unprocessed SIM and SOL. A representative example of SEMs of SD10 and SD30 along with SIM and SOL are presented in Figure 5.

The morphology of SOL (Figure 5a) appeared mostly as a sphere-like mass with a rough surface texture, whereas SIM appeared as small-to-large, irregularly sized and rectangular crystals with an affinity to self-agglomerate. The micrographs for the SDs (Figure 5c,d) showed fractured and irregularly shaped large agglomerates without any visible signs of drug crystals. The absence of drug crystals suggests incorporation of SIM in the polymer in the amorphous form.

### 2.5. In Vitro Dissolution Studies

**Figure 6:**
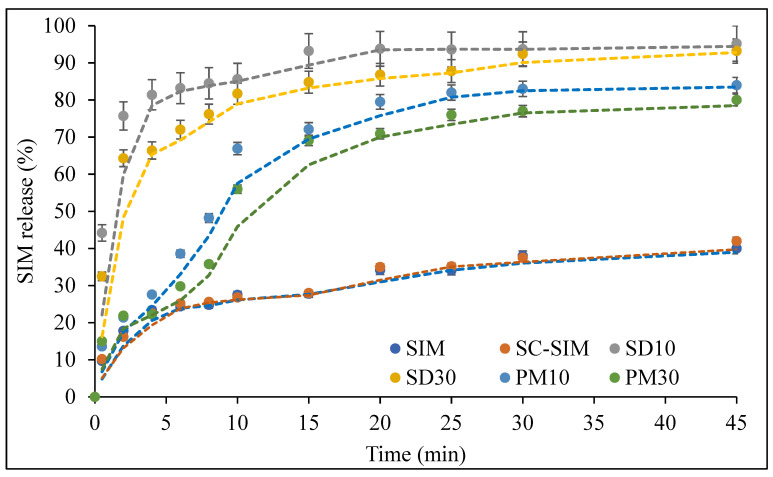
Dissolution profiles of bulk SIM, PMs, and SDs at 37 °C in pH 7 phosphate buffer containing 0.2% *w/v* SDS (*n* = 3).

The drug dissolution profiles of bulk SIM, SC–SIM, PMs, and SDs are presented in Figure 6. The dissolution studies were conducted only on SDs and PMs prepared with 10 and 30% *w/w* SIM, to understand the effect of completely amorphous and partly crystalline systems on the drug dissolution.

scCO_2_ processing of the drug alone at 50 °C and 150 bar did not result in any changes to its dissolution profile. This agrees with the XRD and DSC data, where no changes in crystalline structure of the drug were observed.

**Figure 7:**
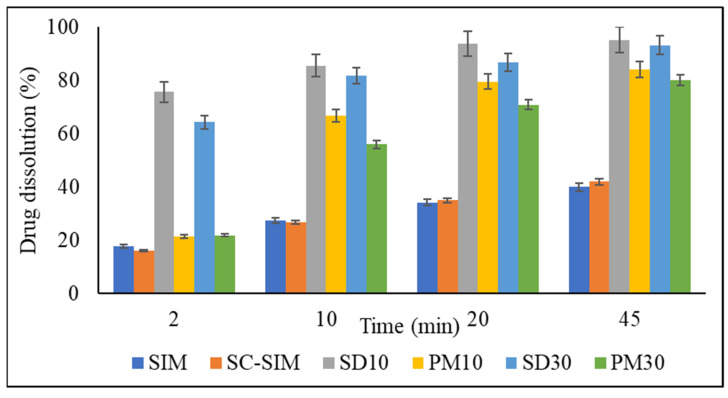
Drug dissolution of pure SIM, scCO_2_ processed SIM, SD, and PM after 2, 10, 20, and 45 min.

The presence of SOL in PMs itself was able to improve drug dissolution when compared to bulk SIM. The drug dissolution from PMs increased steadily with time and resulted in almost 90% SIM release after 45 min compared to 41% of the drug alone. However, scCO_2_-processed SDs resulted in rapid drug dissolution varying from 65 to 75% within the first 2 min and above 80% within 10 min (Figure 7). The drug release from SD10 formulation was ~100% after 45 min. The dissolution profiles of the prepared dispersions showed significant enhancement in the release of SIM in comparison to bulk SIM, and corresponding PMs.

The increased dissolution rates of SDs compared to PMs are related to the formation of a glassy solution during the scCO_2_ processing. The dissolution of CO_2_ in polymeric matrix resulted in enhanced polymer plasticization leading to the solubilisation of SIM within it, as evidenced by the lack of crystallinity in formulated SDs [50,51,52]. The amorphous systems (SDs) offered a lower thermodynamic barrier to dissolution, and a higher internal energy and superior molecular motion that allowed for a faster dissolution rate [45,50].

In general, the higher polymer content in SD or PM resulted in comparatively higher and faster drug dissolution. This can be explained by the hydrophilicity of SOL combined with amorphization of the drug during SD formation. The reduction in interfacial tension between dissolution media and drug due to the presence of SOL results in higher drug dissolution, even from PMs. However, drug amorphicity promoted this further in SDs prepared with scCO_2_ processing. The dissolution of hydrophobic drug from complexes in hydrophilic carriers is known to be governed by the minor (SIM in this case) and typically hydrophobic component, where an increase in the drug content in the formulation increases overall lipophilicity of the matrix, leading to a decrease in percent release and dissolution rate [53,54]. Furthermore, the improved dissolution could also be attributed to the micellar solubilization properties of SOL [55,56,57]. The critical micelle concentration (CMC) of SOL is known to be 7 × 10^−4^% *w/v* at 37 °C [40]. Therefore, the concentration of SOL in all the drug–polymer formulations was above the CMC. The higher and faster drug dissolution from the 10% SD and PM could be attributed to the difference in polymeric micellar concentration, i.e., SD with 10% drug will have a higher concentration of micelles in comparison to 30% SD that can consequently influence the SIM dissolution from these formulations [55]. Nonetheless, the data presented herein show that scCO_2_ can be an efficient green processing method to load CO_2_-philic drugs into polymers such as SOL to achieve improved dissolution rate. 

Kinetic evaluation of drug release profiles from SD10 and SD30 was also performed to understand the mechanism associated with the drug release from these formulations. The release profiles from different SDs were fitted to most common kinetic models, including zero-order, first-order, Higuchi, Hixson–Crowell, and Korsmeyer–Peppas models. The calculated correlation coefficients (R^2^) obtained for various models along with release exponent (*n*) values for the Korsmeyer–Peppas model are presented in Table 3.

Table 3 indicates that the drug release from these formulations followed the Korsmeyer–Peppas model with the R2 values of 0.9566 and 0.9645 for SD10 and SD30, respectively. The values of diffusional exponent (*n*) were calculated to be 0.123 and 0.174 for both solid dispersions. The ‘n’ relates to the mechanism of drug release, i.e., *n* < 0.5 indicates Fickian diffusion; 0.5 < *n* < 0.9, non-Fickian transport (anomalous transport); and *n* > 0.9, type-II transport [58]. The *n* values obtained for both SDs were less than 0.5, suggesting that the mechanism of drug release from these formulations was predominantly diffusion-controlled.

The drug release profiles presented herein provide a good overview of the impact of drug/polymer ratio and processing conditions on the release of SIM from formulated SDs. However, the release was studied only in pH 7 buffer, so it will be appropriate to conduct further studies in simulated gastric and intestinal conditions in the future to understand the influence of variable gastrointestinal conditions on the drug release and their suitability as a potential oral drug delivery system for SIM.

## 3. Materials and Methods

### 3.1. Materials

Simvastatin was supplied by Biocon Limited, India, while Soluplus^®^ was kindly provided by BASF chemical company, Ludwigshafen, Germany. Liquid CO_2_ (99.9%) was supplied by BOC Ltd., Guildford, UK. Chemicals used in this work were of analytical grade and used without any further purification. The buffer for release studies was prepared with deionized water.

### 3.2. Preparation of SIM–SOL SDs

The plasticizing effect of scCO_2_ on SOL is already known in literature [39,59]. Hence, preliminary phase-change studies were carried out only on SIM between 100 and 200 bar. The phase-change studies were performed using SFT Phase monitor II (Supercritical Fluid Technologies Inc., Newark, DE, USA), the detailed schematics of which are presented by Trivedi et al. [60]. For the study, 1–3 mg of SIM was accurately weighed and filled in the melting point capillary prior to placing it in the sample holder. It was tightly screwed onto the high-pressure vessel, which was then filled with liquid CO_2_ to achieve the desired pressure. The pressure in the vessel was kept constant throughout the experiment by adjusting the manually operated piston on the instrument. Experiments were performed with the gradual increase in temperature in increments of 0.2 °C until a phase transition (solid to liquid) at a given pressure was observed, as monitored by a CCD camera attached to a quartz window on the vessel.

#### 3.2.1. Physical Mixtures

SIM and polymer physical mixtures were prepared with 10, 20, and 30% *w/w* drug. The appropriate quantities of SIM and SOL to obtain 5 g of physical mixture (PM) were accurately weighed and added to a pestle and mortar for mixing. Thereafter, the drug and polymer mixtures were gently mixed for 10 min to obtain even distribution of the drug in the polymer. The prepared samples were stored in glass vials at room temperature (23 ± 2 °C) and away from direct sunlight up until further analysis.

#### 3.2.2. Solid Dispersion via scCO_2_ Processing

Following the confirmation of phase change of drug in scCO_2_ at 50 °C/120 bar, and the ability of scCO_2_ to plasticize SOL, the SDs were prepared as follows (Table 4) to investigate the effect of temperature, pressure, duration, and drug/polymer ratio. The experiments were designed using 3 × 2 × 2 × 2 factorial design as follows.

The scCO_2_ processing was carried out in the static mode to obtain SDs using an apparatus supplied by Thar Process Inc., Pittsburgh, PA, USA, as described in detail elsewhere [61]. Next, 2 g of each PM were placed in a high-pressure vessel pre-heated to 40 or 50 °C (±2 °C). The vessel was then closed, and liquid CO_2_ was pumped at a rate of 15 g/min until the required pressure was achieved. The temperature and the pressure were maintained for a pre-determined duration under stirring to promote the phase change and dissolution of drug in the polymer. At the end of experiment, the vessel was depressurised at a rate of 7 bar/minute by venting the CO_2_ in isothermal conditions. The prepared samples were stored in glass vials at room temperature and away from direct sunlight up until further analysis.

### 3.3. Analysis of the Prepared Solid Dispersions

#### 3.3.1. Differential Scanning Calorimetry (DSC) Analysis

DSC analysis of SIM, SOL, SC–SOL, SC–SIM, PMs and SDs was carried out using a DSC823e instrument (Mettler-Toledo, LLC, Leicester, UK) under constant flow of nitrogen. For each sample, approximately 3 mg to 4 mg were accurately weighed and hermetically sealed in aluminium crucibles. The sealed pans were then placed in the DSC sample holder and heated at a rate of 10 °C per minute. The thermogram was collected over the temperature range of 20 °C to 200 °C.

#### 3.3.2. X-ray Diffraction (XRD) Analysis

The crystallinity of SIM, SOL, scCO_2_-processed polymer and drug, PMs and SDs were examined by performing XRD analysis using a Bruker D8 Advance (Bruker, Germany) diffractometer in theta–theta reflection mode with copper anode. Each sample was scanned from 2° to 60° at a step size of 0.02° in the 2θ range. Data collection and interpretations were performed using DiffracPlus and the EVA V.14 program, respectively.

#### 3.3.3. Attenuated Total Reflectance-Fourier Transform Infrared (ATR-FTIR) Spectroscopy

The ATR-FTIR spectra of drug, polymer, scCO_2_-processed polymer and drug, PMs and SDs were obtained using a Spectrum Two FTIR spectrometer (Perkin Elmer, UK). The sample was spread evenly on the surface of a single reflection horizontal ATR accessory with a zinc selenide (ZnSe) crystal. The spectra were collected from the 4000–450 cm^−1^ range in transmission mode. Approximately 16 scans were collected for each spectrum with a resolution of 4 cm^−1^**.**

#### 3.3.4. Scanning Electron Microscopy (SEM)

SEM was carried out in order to determine the shape and surface morphology of the scCO_2_-processed SDs. SEM analysis was also performed on SIM and SOL for comparative purposes. Approximately 1 mg of sample was fixed on a stub with carbon adhesive and the loose particles were removed. Micrographs were then collected after chromium coating of the samples using a Hitachi SU8030 (Hitachi High-Technologies, Maidenhead, UK) scanning electron microscope at a voltage of 1.0 kV.

### 3.4. In Vitro Dissolution Studies

The degree of dissolution of SIM from the PMs and SDs, and for SIM alone, was evaluated using the USP Type II paddle method (Hanson G2 Vision^®^ Classic 6, Chatsworth, Los Angeles, CA, USA) with sodium phosphate buffer (0.01 M, pH 7) containing 0.2% *w/v* Sodium Dodecyl Sulphate (SDS) as the dissolution medium [18,38]. The samples equivalent to 20 mg of SIM were dispersed into 900 mL of the dissolution buffer maintained at 37 ± 0.5 °C and stirred at 50 rpm. At the specified time intervals of 0.5, 2, 4, 6, 8, 10, 15, 20, 25, 30, and 45 min, 5 mL aliquots of the dissolution media were removed and replaced with an equal volume of fresh buffer. The withdrawn sample was filtered and analysed by ultraviolet-visible spectroscopy (Cary 100 UV-Vis spectrometer, Agilent Technologies, Cheadle, UK) at 238 nm to determine the amount of released SIM. These experiments were performed in triplicate.

## 4. Conclusions

In the current study, a solvent-free scCO_2_-based method was employed for the development of amorphous solid dispersions to improve the dissolution rate of simvastatin. The process was introduced as an alternative approach to existing technologies that facilitates the formation of SDs of water insoluble APIs processed at low temperatures and thus preventing drug degradation. Physiochemical characterization of the processed sample demonstrated the formation of glass solution and the presence of SIM in a molecularly dispersed state, even at high drug loadings. The scCO_2_-formed SDs presented very fast dissolution rates with 100% SIM being released within 45 min. Overall, scCO_2_ processing of drug–polymer blends was proved to be an efficient approach for the development of amorphous SDs and can be further used especially for temperature-labile drug substances to prevent possible drug degradation.

## Figures and Tables

**Table 1:** Examples of solid dispersions prepared via scCO_2_ processing of drugs and polymers.

Drug	Polymer/s	T (°C)	P (bar)	Co-Solvent	Outcome	Ref
Oxeglitazar	Poloxamer 188 & 407, PEG 8000, PVP K17	35	80	DCM, CHCl_3,_ EtOH	Improved dissolution rate.	[20]
Carbamazepine	PEG 4000	40	70	Acetone	Particle size reduction.	[21]
Ibuprofen	PVP	35–45	80–220	-	Amorphous drug dispersion in polymer matrix.	[22]
Ibuprofen	Kollidon CL-SF	40	250	-	Amorphous drug in SD. Improvement in in vitro and in vivo performance.	[23]
Silymarin	PVP- K17 & K30, HPMC- K4M & K15M	50	150	EtOH & DCM + EtOH	Drug dispersion in amorphous state. Improved drug dissolution and oral absorption.	[24]
Felodipine	HPMC, Poloxamer 188 & 407, HCO-60	45	100	EtOH & methylene chloride	Drug amorphisation. Improved dissolution (>90% in 2 h).	[25]
Sirolimus	PVP-K30, SLS, TPGS, Sucroester 15, Gelucire 50/30, Myrj 52	40	120	EtOH or DCM + EtOH	Enhanced drug supersaturation, dissolution, stability, and oral bioavailability.	[26]
Cefuroxime axetil	HPMC 2910/PVP-K30	45	100	DCM + EtOH	Drug in amorphous form due to intermolecular H-bonds between drug and polymers.	[27]
Indomethacin	PVP	35	85	Acetone + DCM	Drug amorphisation. Impact of polymer content.	[28]
Telmisartan	HPMC + PVP	45	120	EtOH + methylene chloride	Drug in amorphous state but prone to re-crystallization.	[29]
Lactulose	Chitosan scaffolds/microspheres	60, 100	100	EtOH + Water	Drug impregnation of chitosan with mono-or disaccharides.	[30]
Nimesulide	HPMC + PVP	40	80	DCM + MeOH	Complete amorphisation of drug. Increased drug solubility of >5 folds.	[31]
Furosemide	Crospovidone	39.85	100, 200	Acetone	Amorphous SD, with stability >6 months.	[32]
Glibenclamide	HPMCE5, PEG6000, Poloxamer 407	50	103–206	Acetone, methanol	Drug amorphisation. Improved dissolution.	[33]
Nimodipine	PVP K-30	40	100	-	Long processing time (3 days). Drug amorphisation and improved dissolution.	[34]
Tacrolimus	Soluplus, Chitosan, PVP, HPMC, TPGS	40	100–300	-	Best SDs with Soluplus with improved drug dissolution.	[35]

**Table 2:** Solid dispersion of simvastatin in Soluplus via scCO_2_ processing.

Drug Content (% *w/w*)	Temperature (°C)	Pressure (bar)	Duration (min)	Observation (Drug Form)
10	40	100	60	Crystalline
	40	100	120	Crystalline
	50	100	60	Crystalline
	50	100	120	Crystalline
	40	150	60	Crystalline
	40	150	120	Crystalline
	50	150	60	Semi-crystalline
	50	150	120	Amorphous
20	40	100	60	Crystalline
	40	100	120	Crystalline
	50	100	60	Crystalline
	50	100	120	Crystalline
	40	150	60	Crystalline
	40	150	120	Crystalline
	50	150	60	Semi-crystalline
	50	150	120	Amorphous
30	40	100	60	Crystalline
	40	100	120	Crystalline
	50	100	60	Crystalline
	50	100	120	Crystalline
	40	150	60	Crystalline
	40	150	120	Crystalline
	50	150	60	Semi-crystalline
	50	150	120	Semi-crystalline *

* Semicrystalline likely due to the high drug content.

**Table 3:** Correlation coefficients (R^2^) and release exponent (*n*) values obtained from different kinetic models for drug release from SDs.

Kinetic Model	SD10	SD30
Zero-order	0.5844	0.6839
First-order	0.8577	0.7796
Higuchi	0.3322	0.3239
Hixson–Crowell	0.4657	0.3612
Korsmeyer–Peppas	0.9566	0.9645
Korsmeyer–Peppas (*n*)	0.123	0.174

**Table 4:** Experimental design for the preparation of Simvastatin–Soluplus solid dispersions via scCO_2_ processing.

Factor	Name (Unit)	Low	Mid	High
A	Drug (%)	10	20	30
B	Temperature (°C)	40	-	50
C	Pressure (bar)	100	-	150
D	Time (min)	60	-	120

## Data Availability

Data are contained within the article.
